# Dietary Crocin Inhibits Colitis and Colitis-Associated Colorectal Carcinogenesis in Male ICR Mice

**DOI:** 10.1155/2012/820415

**Published:** 2012-12-25

**Authors:** Kunihiro Kawabata, Nguyen Huu Tung, Yukihiro Shoyama, Shigeyuki Sugie, Takayuki Mori, Takuji Tanaka

**Affiliations:** ^1^Division of Palliative Care and Department of Internal Medicine, Tokai Central Hospital, 4-6-2 Sohara-Higashijima-cho, Kakamigahara 504-8601, Japan; ^2^Department of Pharmacognosy, Faculty of Pharmaceutical Sciences, Nagasaki International University, 2825-7 Huis Ten Bosch-cho, Sasebo 859-3298, Japan; ^3^Department of Pathology, Murakami Memorial Hospital, Asahi University, 3-23 Hashimoto-cho, Gifu 500-8523, Japan; ^4^Department of Pharmacy, Ogaki Municipal Hospital, 4-86 Minaminokawa-cho, Ogaki 503-8502, Japan; ^5^Division of Cytopathology, The Tokai Cytopathology Institute: Cancer Research and Prevention (TCI-CaRP), 5-1-2 Minami-Uzura, Gifu 500-8285, Japan; ^6^Department of Tumor Pathology, Graduate School of Medicine, Gifu University, Gifu 501-1194, Japan

## Abstract

A natural carotenoid crocin is contained in saffron and gardenia flowers (crocuses and gardenias) and is used as a food colorant. This study reports the potential inhibitory effects of crocin against inflammation-associated mouse colon carcinogenesis and chemically induced colitis in male ICR mice. In the first experiment, dietary crocin significantly inhibited the development of colonic adenocarcinomas induced by azoxymethane (AOM) and dextran sodium sulfate (DSS) in mice by week 18. Crocin feeding also suppressed the proliferation and immunohistochemical expression of nuclear factor- (NF-) **κ**B but increased the NF-E2-related factor 2 (Nrf2) expression, in adenocarcinoma cells. In the second experiment, dietary feeding with crocin for 4 weeks was able to inhibit DSS-induced colitis and decrease the mRNA expression of tumor necrosis factor **α**, interleukin- (IL-) 1**β**, IL-6, interferon **γ**, NF-**κ**B, cyclooxygenase-2, and inducible nitric oxide synthase in the colorectal mucosa and increased the Nrf2 mRNA expression. Our results suggest that dietary crocin suppresses chemically induced colitis and colitis-related colon carcinogenesis in mice, at least partly by inhibiting inflammation and the mRNA expression of certain proinflammatory cytokines and inducible inflammatory enzymes. Therefore, crocin is a candidate for the prevention of colitis and inflammation-associated colon carcinogenesis.

## 1. Introduction

A perennial stemless herb* Crocus sativus* L. (Iridaceae), commonly known as saffron, is widely cultivated worldwide, especially in Iran, India, Greece, Morocco, Spain and China. Saffron has several biological activities and is used in folk medicine [1, 2]. The major biologically active ingredient in saffron is known to be crocin, which is an ester glycoside of crocetin.

The other typical components are picrocrocin and safranal, which are related to the flavor of the herb [1–4]. Pharmacological studies have reported that saffron extracts and/or the active constituents have properties that improve learning and memory [5, 6], as well as anticonvulsant [7], antidepressant [8], antiinflammatory [9, 10], and antitumor effects [1, 2]. Free radical scavenging, antioxidant activity, and the promotion of the diffusion of oxygen in different tissues were also reported for saffron extracts or their bioactive constituents [11–13]. Other biological effects of saffron and its constituents include the induction of apoptosis [14, 15], antihyperlipidemic effects [16], immuno modulation [17], and anti-neurodegenerative effects [18–20]. Our previous studies on saffron and/or crocetin glycosides indicated the prevention of skin tumor promotion in mice [21] and the decrease in the proliferation of human colorectal cancer (CRC) cells [22].

With regard to the effects of saffron and its active ingredients on carcinogenesis, many *in vitro* studies have demonstrated that extracts of saffron and certain components of the herb are able to inhibit the growth of several types of human cancer cells [14, 23–25], including CRC cells, as we reported in a previous study [22]. However, there have so far been few *in vivo* studies conducted to demonstrate the anticancer effects of saffron and its constituents [26–30]. 

Patients with ulcerative colitis (UC) and Crohn's disease, two major types of inflammatory bowel disease (IBD), are at high risk of developing CRC [31–33]. Unlike sporadic CRC, the CRC in UC patients arises from focal or multifocal dysplastic crypts that are present in areas of inflammation [32]. Growing evidence supports a significant role for several cytokines produced by epithelial and immune cells, in the pathogenesis of IBD-related CRC [34]. To investigate the pathobiology of IBD-related CRC, we developed a colitis-associated and two-stage mouse CRC model [35]. Using this model that mimics human CRC in the inflamed colon [35, 36], we have reported several synthetic and natural compounds which effectively suppressed colitis-associated colon carcinogenesis [37–40]. 

**Figure 1:**
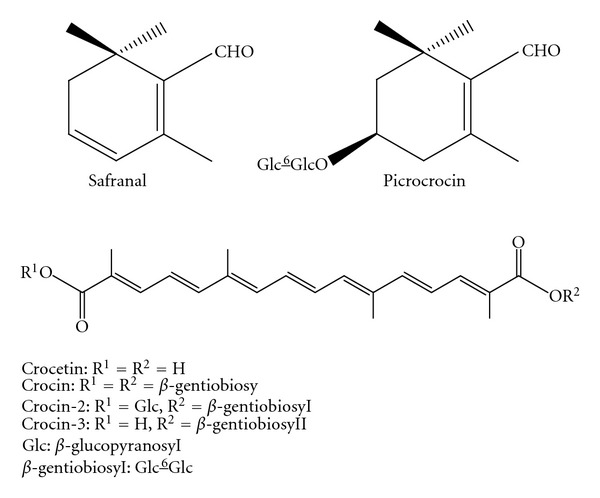
Structures of the principle constituents (crocetin, crocetin-diglycoside, crocetin-triglycoside, crocin, picrocrocin, and safranal) of saffron.

Nuclear factor (NF-) *κ*B is a well-established regulator of genes encoding cytokines, cytokine receptors, and cell adhesion molecules that drive immune and inflammatory responses [41]. Recently, NF-*κ*B activation has also been connected with multiple aspects of oncogenesis and NF-*κ*B is one of the potential targets of anticancer agents [42]. NF-*κ*B regulates the expression of several genes, such as cyclooxygenase- (COX-) 2, inducible nitric oxide synthase (iNOS), tumor necrosis factor (TNF-) *α*, interleukin (IL-) 1*β*, cell surface adhesion molecules, and antiapoptotic proteins, which are involved in tumor initiation, promotion, and metastasis [43]. Therefore, NF-*κ*B has become one of the most important targets for cancer chemoprevention [44]. Since crocetin has been reported to inhibit chemically-induced colitis in mice by down regulation of NF-*κ*B [10], crocin, a glycoside of crocetin (see Figure 1), may also inhibit colitis and colitis-associated CRC by affecting inflammatory phenomena. The production of interferon (IFN-) *γ* has been reported in human IBD and IBD-related CRC [45] and experimental UC-associated CRC [46]. A protective role for NF-E2-related factor 2 (Nrf2) against the toxicity of xenobiotics has been suggested [47, 48], making it one of the targets for cancer chemoprevention [47–50]. Therefore, these factors can be used to assess the effects of molecules against inflammation and cancer.

The aim of this study was to investigate the possible inhibitory effects of crocin isolated from saffron against colitis-associated colon carcinogenesis using an AOM/DSS mouse model. This study contained two different experiments. In the first experiment, we evaluated the effects of three different concentrations (50, 100, and 200 ppm) of crocin in the diet on colitis-associated colorectal carcinogenesis in mice. In addition, the immunohistochemical expression of NF-*κ*B and Nrf2 [49–51] in adenocarcinoma cells was examined. The second experiment was conducted to determine the effects of these concentrations of crocin on DSS-induced colitis and the mRNA expression of NF-*κ*B, IFN-*γ*, TNF-*α*, IL-1*β*, IL-6, COX-2, iNOS, and Nrf2 in mice, since the elevated mRNA expression of pro-inflammatory cytokines and inducible inflammatory enzymes caused by inflammatory stimuli plays a significant role in carcinogenesis [38]. 

## 2. Materials and Methods

### 2.1. Animals, Chemicals, and Diet

Male Crj: CD-1 (ICR) mice (Charles River Japan, Inc., Tokyo) aged 4 weeks were used in these studies. They were maintained in the BioGate Inst., Co., Ltd., (Yamagata City, Gifu 501-2123, Japan), according to the Institutional Animal Care Guideline. All animals were housed in plastic cages (4 or 5 mice/cage) with free access to drinking water and a basal diet, CE-2 (CLEA Japan Inc., Tokyo, Japan), under controlled conditions of humidity (50 ± 10%), light (12/12 h high/dark cycle), and temperature (23 ± 2°C). After arrival, animals were quarantined for the first seven days and randomized by body weights into the experimental and control groups. AOM and DSS (with a molecular weight of 36,000–50,000, Cat. no. 160110) were purchased from Sigma Chemical Co. (St. Louis, MO, USA) and MP Biomedicals, LLC (Aurora, OH, USA), respectively. DSS was dissolved in water at a concentration of 1.5% (w/v) to induce colitis. The experiments and study designs were approved by the Institutional Committee. All handling and procedures were carried out in accordance with the appropriate Institutional Animal Care Guidelines.

### 2.2. Purification of Crocin

Crocin (purity ≥ 96% by HPLC) was purified from the stigmas of *C. sativus* as reported previously [52]. Briefly, the air- and shade-dried saffron (500 g) was extracted with 50% EtOH (2.0 l × 3 times) at 40°C under sonication. The combined extracts were concentrated to produce a dark-brown syrup (280 g). A part of the obtained crude extract (105 g) was suspended in water (500 mL), then partitioned with CH_2_Cl_2_ (500 mL × 3), and the water layer was subjected to a Diaion HP-20 column elution with a stepwise gradient of MeOH-H_2_O (25, 50, 75, and 100% MeOH; v/v) to afford four fractions (fr. 1.1–1.4). Fr. 1.3 (12.5 g) was subjected to a reversed-phase column with MeOH-H_2_O (3 : 4, v/v) to give five fractions (fr. 4.1–4.4). Fr. 4.2 (3.2 g) was then repeatedly separated over a reversed-phase column with MeOH-H_2_O (1 : 1, v/v) to yield crocin (2100 mg). The structure of crocin was confirmed by NMR and mass spectrometry. 

### 2.3. Experiment 1 (18-Week Study)

**Figure 2:**
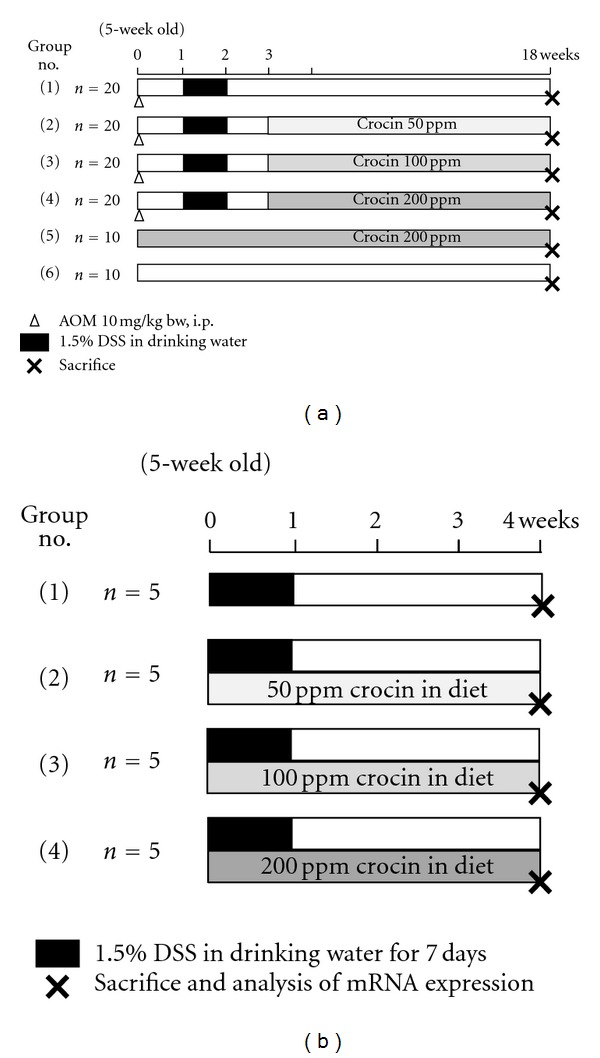
Experimental protocols for (a) Experiment 1 (18-week study) and (b) Experiment 2 (four-week study).

A total of 100 male ICR mice were divided into five experimental and control groups (Figure 2(a)). Mice in groups 1–4 were given a single intraperitoneal injection of AOM (10 mg/kg body weight). Starting one week after the injection, they received 1.5% DSS in their drinking water for seven days. Subsequently, groups 1 (*n* = 20), 2 (*n* = 20), 3 (*n* = 20), and 4 (*n* = 20) received diets containing 0, 50, 100, and 200 ppm crocin for 15 weeks, respectively, starting one week after cessation of DSS exposure. Group 5 (*n* = 10) not treated with AOM or DSS and was fed the 200 ppm crocin-containing diet for 18 weeks. Group 6 (*n* = 10) was served as an untreated control. All animals were sacrificed at week 18 by an overdose of ether to determine the effects of crocin on colon tumorigenesis. At the time of sacrifice, complete necropsies were done on all mice. The entire body, liver, and kidneys were weighted, and then liver and kidneys were fixed in 10% buffered formalin for 24 h. After macroscopic inspection, tissues from the large bowel, liver, and kidneys were processed for histopathological examination by conventional methods. The histopathological examination was performed on paraffin-embedded sections after hematoxylin and eosin (H&E) staining.

### 2.4. Experiment 2 (Four-Week Study)

A total of 20 male ICR mice were divided into four experimental and control groups and subjected to a four-week experiment (Figure 2(b)). Mice in groups 1 through 4 were fed the experimental diets containing 0, 50, 100, and 200 ppm crocin, respectively, for four weeks. During the first week of the experiment, all groups were given 1.5% DSS in their drinking water. All animals were sacrificed at week four and their large bowels were flushed with saline and then excised. After measurement of their length (from the ileocecal junction to the anal verge), they were cut open longitudinally along the main axis, and gently washed with saline to remove feces. The large bowel was macroscopically inspected for the presence of pathological lesions, including ulcerations and cuts and fixed in 10% buffered formalin for 24 h. A histopathological examination was performed on paraffin-embedded sections from the large bowel after H&E staining to determine the inflammation score of the colonic mucosa.

### 2.5. Scoring Inflammation in the Colorectum

The inflammation in the large bowel was scored on the H&E-stained sections. Large intestinal inflammation was graded according to the morphological criteria described in our previous study [53]: grade 0, normal appearance; grade 1, shortening and loss of the basal 1/3 of the actual crypts with mild inflammation in the mucosa; grade 2, loss of the basal 2/3 of the crypts with moderate inflammation in the mucosa; grade 3, loss of all of the crypts with severe inflammation in the mucosa and submucosa, while retaining the surface epithelium; grade 4, the presence of mucosal ulcer with severe inflammation (infiltration of neutrophils, lymphocytes, and plasma cells) in the mucosa, submucosa, muscularis propria, and/or subserosa. The scoring was performed on the entire colon with or without proliferative lesions and was expressed as a mean score/mouse. 

### 2.6. Immunohistochemistry of Minichromosome Maintenance Protein 2 (MCM2), NF-*κ*B, and Nrf2 in Adenocarcinomas

We used 4 *μ*m thick paraffin-embedded sections from the colons of the mice in all groups from both experiments for the immunohistochemical analysis using the labeled streptavidin biotin method with an LSAB Kit (DAKO Japan, Kyoto, Japan) and with microwave accentuation. The paraffin-embedded sections were heated for 30 min at 65°C, deparaffinized in xylene, and rehydrated through graded ethanol solutions at room temperature. A Tris-HCL buffer (0.05 M, pH 7.6) was used to prepare solutions to rinse slides between the various steps. Incubations were performed in a humidified chamber. The sections were treated for 40 min at room temperature with 2% bovine serum albumin and incubated overnight at 4°C with primary antibodies. The primary antibodies used were anti-MCM2 rabbit monoclonal antibody (no. 3619, anti-MCM2 (D7611)XP, 1 : 400 dilution; Cell Signaling Technology, Inc., Danvers, MA, USA), anti-NF-*κ*B p50 (H-119) rabbit polyclonal antibody (sc-7178, 1 : 500 dilution; Santa Cruz Biotechnology, Inc., Santa Cruz, CA, USA), and anti-Nrf2 rabbit polyclonal antibody (ab31163, 1 : 500 dilution; Abcam, Inc. Cambridge, MA, USA). These antibodies were applied to the sections according to the manufacturer's protocol. The horseradish peroxidase activity was visualized by treatment with H_2_O_2_ and 3,3′-diaminobenzidine for 5 min. As the final step, the sections were weakly counterstained with Mayer's hematoxylin (Merck, Tokyo, Japan). For each case, the negative controls were examined first in the serial sections without the primary antibodies.

In Experiment 1, an immunohistochemical analysis was done in five mice each from groups 1 through 4. The immunoreactivity against the antibodies was assessed in the colonic adenocarcinomas (>3 mm in diameter) that developed in these groups using a microscope (Olympus BX41, Olympus Optical Co., Tokyo, Japan). The intensity and localization of the immunoreactivity against the primary antibodies were determined by a pathologist (T. Tanaka) who was unaware of the treatment group to which the slide belonged. The number of nuclei with positive reactivity for MCM2 was counted in a total of 3 × 100 cells in three different areas of the colonic cancer and expressed as a percentage (mean ± SD). The immunoreactivity against the NF-*κ*B and Nrf2 antibodies in the adenocarcinoma cells was evaluated and graded between 0 and 5; grade 0, <15% of cells showing positive reactivity; grade 1, 16~30% of cells showing positive reactivity; grade 2, 31~45% of cells showing positive reactivity; grade 3, 46~60% of cells showing positive reactivity; grade 4, 61~75% of cells showing positive reactivity; grade 5, 76%+ of cells showing positive reactivity. 

### 2.7. Total RNA Extraction and Quantitative Real-Time PCR

Total RNA was extracted from the colonic mucosa using the RNeasy Mini Kit (Qiagen, Tokyo, Japan) according to the manufacturer's protocol. The cDNA was then synthesized from total RNA using the High-Capacity cDNA Reverse Transcription Kit (Applied Biosystems Japan Ltd., Tokyo, Japan). A quantitative real-time PCR analysis of individual cDNA was performed with an ABI Prism 7500 instrument (Applied Biosystems Japan Ltd., Tokyo, Japan) using TaqMan Gene Expression Assays (Applied Biosystems Japan Ltd., Tokyo, Japan; IFN-*γ*, Mm00801778_m1Mm00801778_m1; NF-*κ*B, Mm00476361_m1: TNF-*α*, Mm00443258-m1; IL-1*β*, Mm00434228_m1; IL-6, Mm00446190-mL; COX-2 (Ptgs2), Mm00478374-mL; iNOS (Nos2), Mm00440485-mL; *β*-actin: Mm00607939-sl). The sense and antisense primers for Nrf2 mRNA were 5′-TTGGCAGAGACATTCCCAT-3′ and 5′-GCTGCCACCGTCACTGGG-3′, respectively. The PCR cycling conditions were 50°C for 2 min and 95°C for 10 min, followed by 40 cycles of 95°C for 15 s and 60°C for 1 min. The expression level of each gene was normalized to the *β*-actin expression level using the standard curve method. Each assay was performed in triplicate and the average was calculated. 

### 2.8. Statistical Analysis

Measurements of multiplicity of colonic lesions and scores of histology and immunohistochemistry were statistically analyzed using either the Tukey or Bonferroni multiple comparison posttest. The incidences of colonic lesions between the groups were compared by Fisher's exact probability test. The statistical analysis of mRNA expression was performed by the Kruskal-Wallis test. Differences were considered to be statistically significant at *P* < 0.05.

## 3. Results

### 3.1. Experiment 1 (18-Week Study)

#### 3.1.1. General Observations

Feeding the mice with the three different crocin-containing diets did not produce any observable clinical toxicity. This was confirmed by histopathological examinations of the liver and kidneys of the mice (data not shown). The mean weights of the whole body, and liver (g/100 g body weight) and the colon length in all groups at week 18 did not differ significantly among the groups (Table 1).

#### 3.1.2. Incidence and Multiplicity of Severe Inflammation with Mucosal Ulcers and High-Grade Dysplastic Crypts

**Figure 3:**
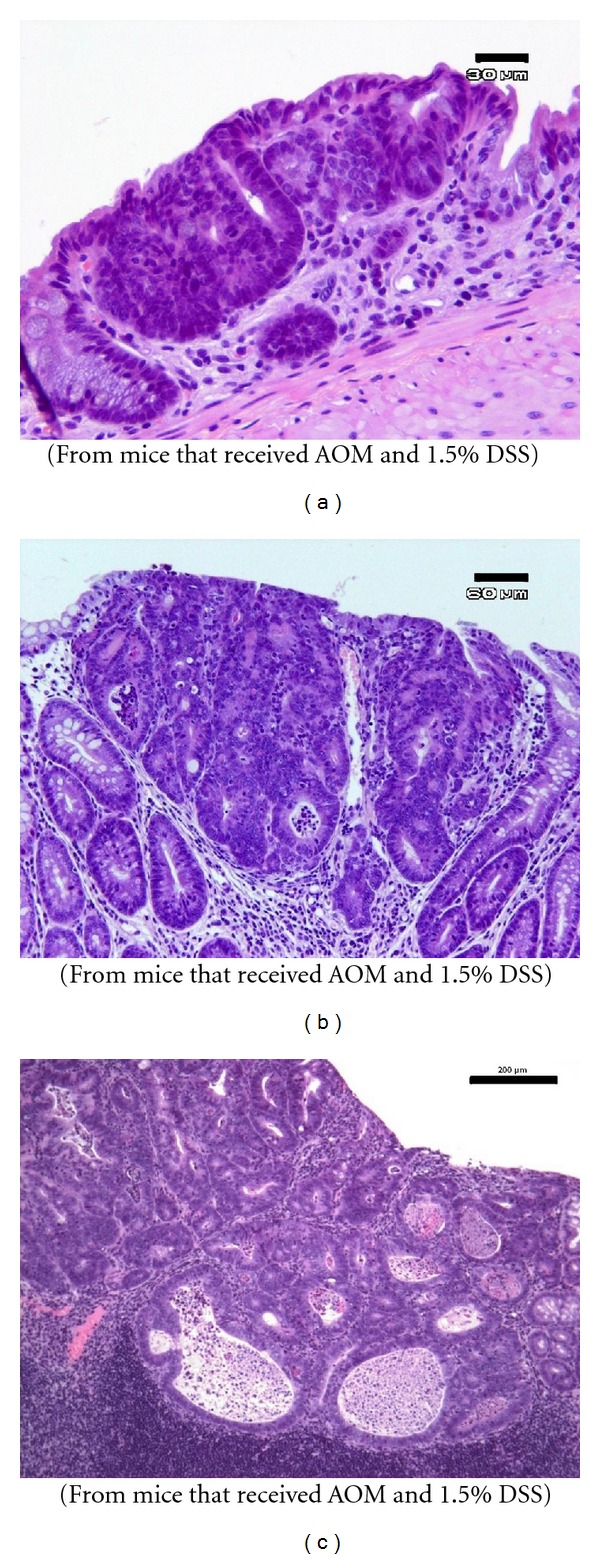
Representative histopathology of colonic proliferative lesions that developed in mice that received AOM and 1.5% DSS (Experiment 1). (a) Dysplastic crypts, high grade (bar, 30 *μ*m); (b) tubular adenomas (bar, 60 *μ*m); (c) tubular adenocarcinoma (bar, 200 *μ*m). H&E stain.

**Figure 4:**
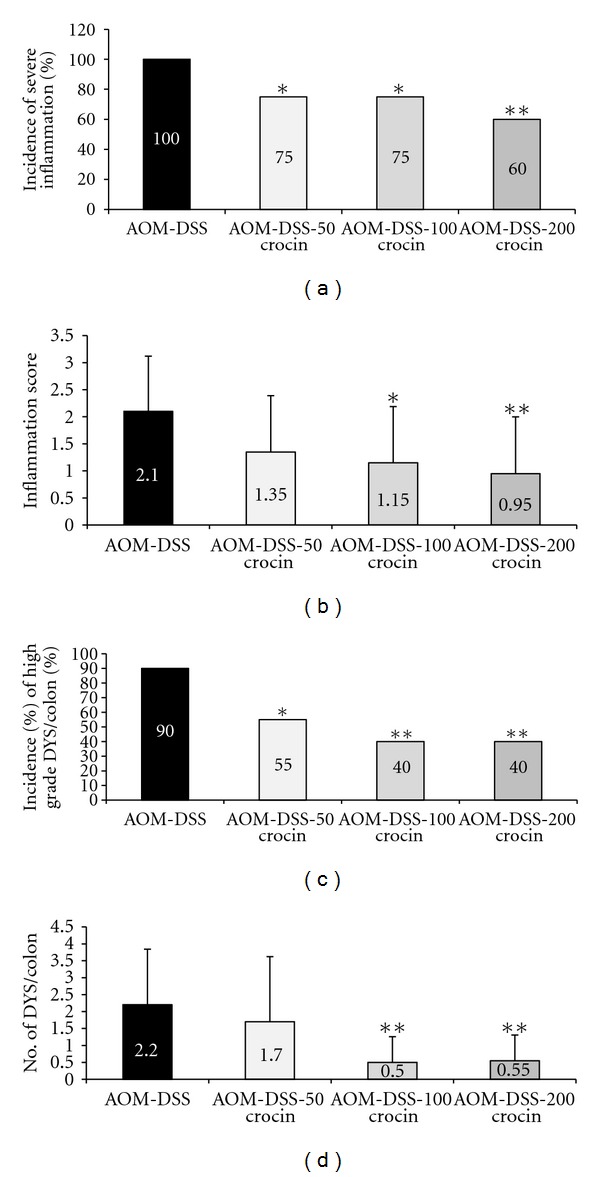
(a) The incidence of severe colorectal inflammation, (b) the inflammation score of colorectum, (c) the incidence of high-grade dysplastic crypts (DYS), and (d) the multiplicity (no./colon) of high-grade DYS. *P < 0.05, **P < 0.01 versus the the AOM + 1.5% DSS group.

AOM and/or DSS treatment resulted in the occurrence of veracious colorectal lesions, such as colitis with mucosal ulcers, dysplastic crypts (high grade, Figure 3(a)), tubular adenoma (Figure 3(b)), and tubular adenocarcinoma (Figure 3(c)). The incidences and multiplicity of severe colorectal inflammation with mucosal ulcers, the inflammation score, and the presence of dysplasia at week 18 are shown in Figure 4. The incidence of severe inflammation with mucosal ulcers (Figure 4(a), *P* < 0.05 or *P* < 0.01) significantly decreased after feeding the mice with all three concentrations of crocin compared with group 1 (AOM + DSS). Similarly, the inflammation score (Figure 4(b), *P* < 0.05 or *P* < 0.01) decreased after crocin treatment at the higher concentrations (100 and 200 ppm). The incidence of high-grade dysplastic crypts (Figure 4(c), *P* < 0.05 or *P* < 0.01) significantly decreased by feeding the mice with all three concentrations of crocin compared with group 1 (AOM + DSS). The multiplicity of high-grade dysplastic crypts (Figure 4(d), *P* < 0.01) also decreased by crocin treatment at the higher concentrations (100 and 200 ppm). 

#### 3.1.3. Incidence and Multiplicity of Colorectal Adenomas and Adenocarcinomas

**Figure 5:**
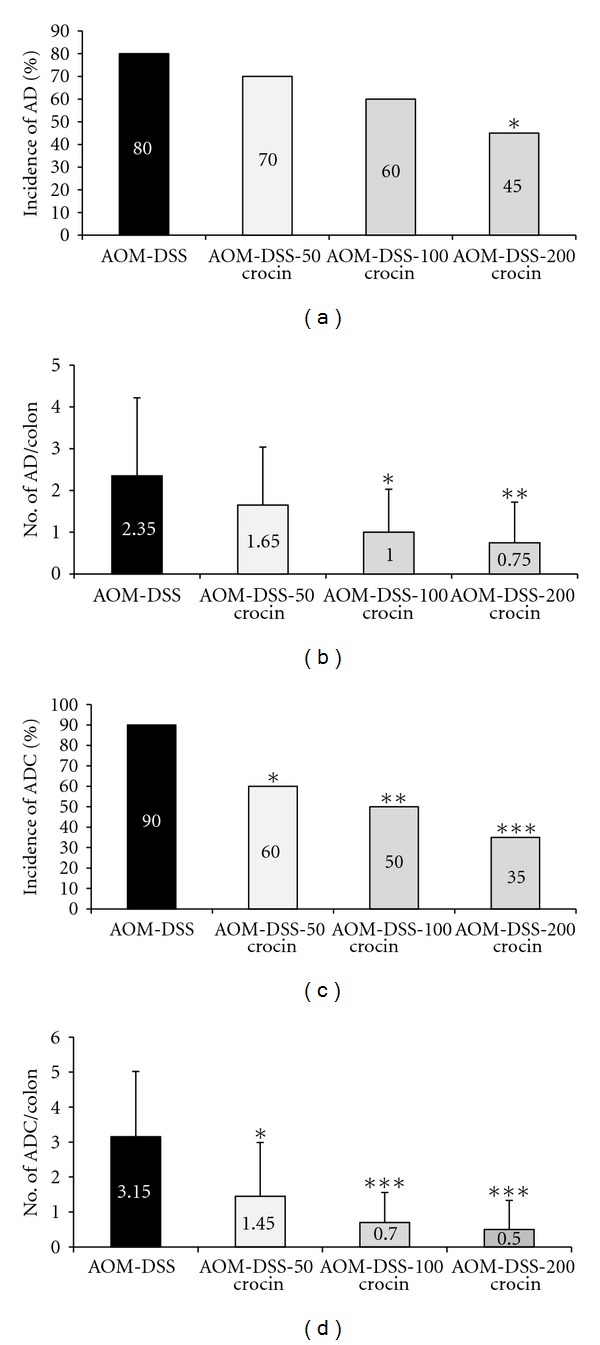
(a) The incidence of colorectal adenoma (AD), (b) multiplicity (no./colon) of colorectal AD, (c) incidence of colorectal adenocarcinoma (ADC), and (d) multiplicity (no./colon) of colorectal ADC. *P < 0.05, **P < 0.01, ***P < 0.001 versus the AOM + 1.5% DSS group.

The incidence and multiplicity of colonic tumors at week 18 are shown in Figure 5. Group 1 (AOM + DSS) had colonic adenocarcinoma with an incidence of 90% and a multiplicity of 3.15 ± 1.87. Treatment with all three concentrations of crocin significantly reduced the incidence (*P* < 0.05 at 50 ppm, *P* < 0.01 at 100 ppm, and *P* < 0.001 at 200 ppm) and multiplicity of adenocarcinoma (*P* < 0.05 at 50 ppm, *P* < 0.001 at 100 ppm and 200 ppm). Dietary crocin also decreased the incidence of adenomas and the difference between groups 1 and 4 was statistically significant (*P* < 0.05). Dietary administration of crocin also decreased the multiplicities of colonic adenoma (*P* < 0.05 at 100 ppm and *P* < 0.01 at 200 ppm).

#### 3.1.4. Cell Proliferation

**Figure 6:**
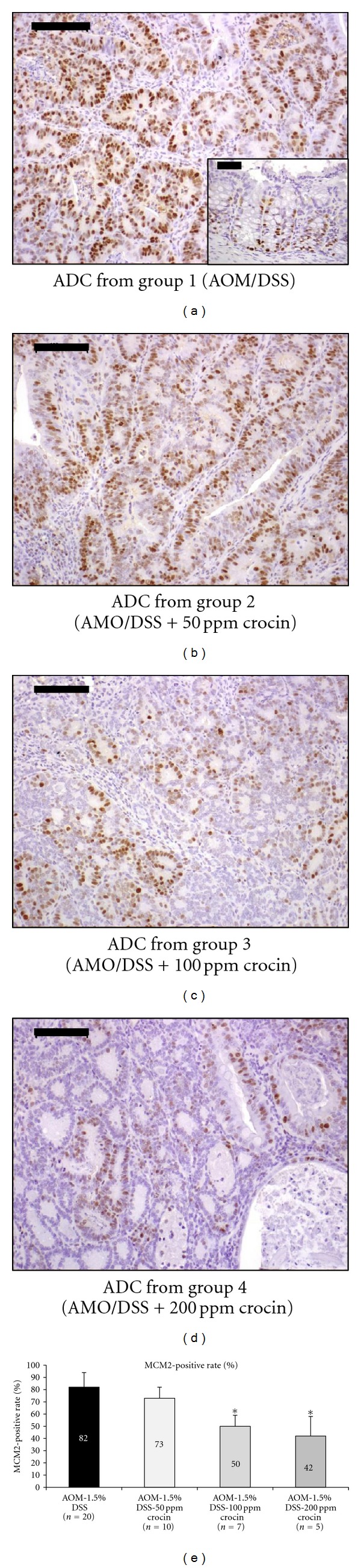
Immunohistochemical staining for MCM2 in an adenocarcinoma that developed in a mouse from (a) group 1 (AOM + 1.5% DSS), (b) group 2 (AOM + 1.5% DSS + 50 ppm crocin), (c) group 3 (AOM + 1.5% DSS + 100 ppm crocin), and group 4 (AOM + 1.5% DSS + 200 ppm crocin). The insert in (a) is normal colonic mucosa. Bars, 100 *μ*m. The graph summarizes the data on the MCM2-positive rates of adenocarcinomas from groups 1 through 4 (*n* = 5 each). *P < 0.001 versus the AOM + 1.5% DSS group.

We immunohistochemically analyzed the expression of MCM2 in colonic adenocarcinomas to determine the effects of crocin on the proliferation of cancer cells (Figures 6(a)–6(d)). As shown in the bar graph in Figure 6(e), the mean MCM2-positive indices of colonic adenocarcinomas in groups 3 (*P* < 0.001) and 4 (*P* < 0.001) were significantly lower than that of group 1, thus indicating that crocin decreased the cancer cell proliferation. 

#### 3.1.5. Immunohistochemical Expression of NF-*κ*B and Nrf2

**Figure 7:**
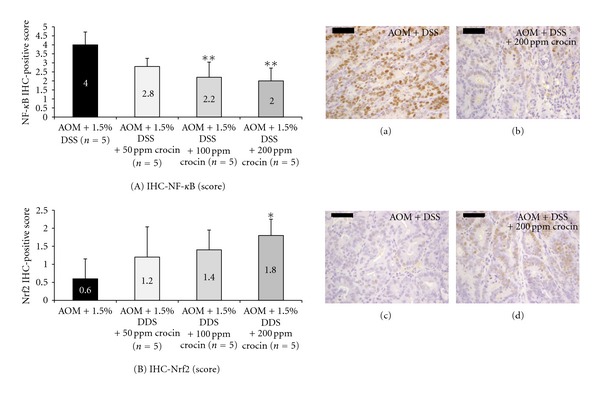
Immunohistochemical expression of (a) NF-*κ*B and (b) Nrf2 in adenocarcinoma cells (Experiment 1). Both proteins were expressed in the nuclei of cancer cells. The scores of immunohistochemical expression of both proteins were changed by crocin treatment: crocin feeding lowered the score for NF-*κ*B (A) and increased it for Nrf2 (B). *P < 0.05, **P < 0.01 versus the AOM + 1.5% DSS group.

Figure 7 shows the immunohistochemical expression of NF-*κ*B (Figures 7(a) and 7(b)) and Nrf2 (Figures 7(c) and 7(d)) in the adenocarcinomas that developed in the colons of the mice from groups 1 and 4. When compared with group 1, the consumption of dietary crocin at 100 ppm (*P* < 0.01) and 200 ppm (*P* < 0.01) in the diet significantly suppressed the immunohistochemical score for NF-*κ*B (Figure 7(a)), while significantly enhancing the expression of Nrf2 at 200 ppm crocin (Figure 7(b), *P* < 0.05). In groups 5 (200 ppm crocin alone) and 6 (untreated), the immunohistochemical expressions of NF-*κ*B and Nrf2 in the colonic mucosa were very weak (data not shown).

### 3.2. Experiment 2 (Four-Week Study)

#### 3.2.1. General Observations

Feeding with experimental diets containing three concentrations (50, 100, and 200 ppm) of crocin did not produce any clinical toxicity. All mice treated with DSS alone (group 1) had diarrhea with bleeding during the DSS treatment. However, fewer mice in groups 2 through 4 had such symptoms. 

#### 3.2.2. Inflammation Scores in the Large Bowel

**Figure 8:**
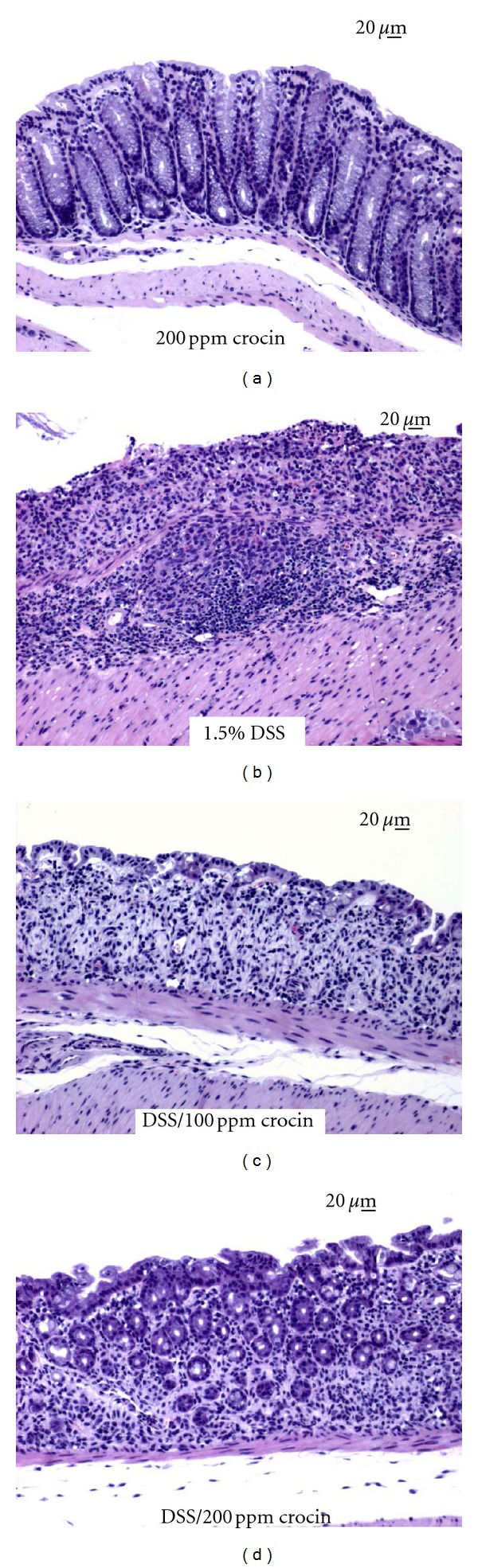
Representative histopathology of the colorectal mucosa (Experiment 2). When compared to (a) normal colorectal mucosa, 1.5% DSS treatment resulted in severe colitis with mucosal ulceration (b). In contrast, mucosal regeneration was observed in the colons of mice that were treated with crocin at (c) 100 ppm and (d) 200 ppm. Bars are 20 *μ*m. H&E stain.

**Figure 9:**
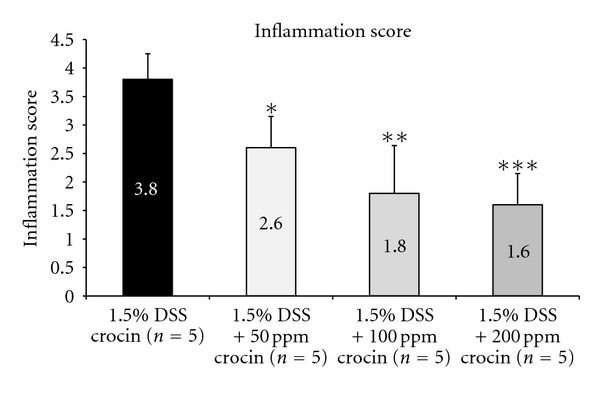
The inflammation scores in the colorectum of mice treated with DSS and or crocin (Experiment 2). Feeding with crocin at all three concentrations (50, 100, and 200 ppm) significantly decreased the inflammation score. *P < 0.05, **P < 0.01, ***P < 0.001 versus the AOM + 1.5% DSS group.

Figure 8 shows the histopathology of the colonic mucosa and summarizes the scoring of colonic inflammation (Figure 9) at week 4. The colonic mucosa of the mice treated with crocin (200 ppm in the diet) alone showed almost normal histology (Figure 8(a)). DSS treatment caused severe colitis with mucosal ulcers (Figure 8(b)). However, the severity of colitis in the mice fed crocin at 100 ppm (Figure 8(c)) and 200 ppm (Figure 8(d)) decreased and regenerative crypt cells covered and healed the mucosal ulcers. As shown in Figure 9, the inflammation scores of the DSS + 50 ppm crocin (*P* < 0.05), DSS + 100 ppm crocin (*P* < 0.01), and DSS + 200 ppm crocin (*P* < 0.001) groups were significantly lower than those of the DSS alone group. 

#### 3.2.3. mRNA Expression Levels of Inducible Inflammatory Enzymes, Proinflammatory Cytokines, and Nrf2

**Figure 10:**
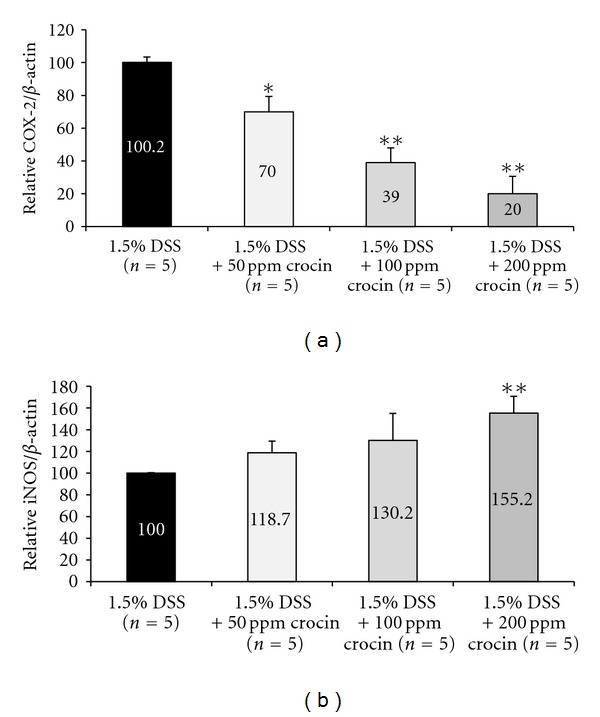
The mRNA expression levels of inducible inflammatory enzymes, (a) COX-2 and (b) iNOS, in the colorectum (Experiment 2) as determined by quantitative real-time RT-PCR. Crocin treatment significantly decreased the expression levels of COX-2 (50, 100, and 200 ppm) and iNOS (200 ppm), when compared with the AOM and DSS group. The expression was normalized to the *β*-actin mRNA expression. Samples were analyzed in triplicate. Data are the means ± SD from three independent assays (*n* = 5 from each group). The ordinates show the relative mRNA expression (/*β*-actin) versus the 1.5% DSS group. *P < 0.01, **P < 0.001 versus the 1.5% DSS group.

**Figure 11:**
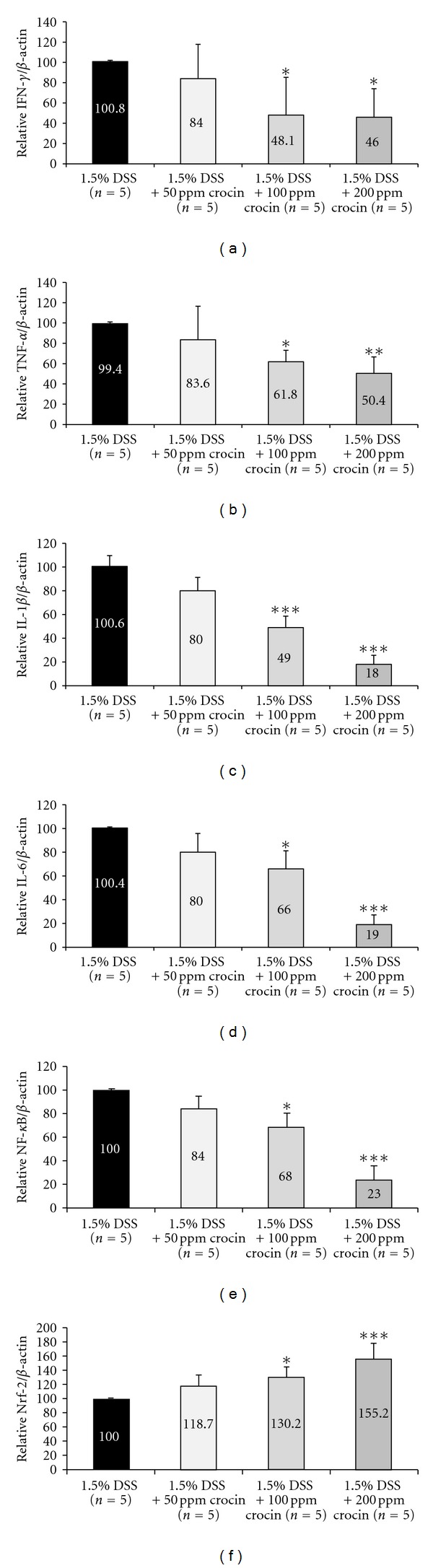
The mRNA expression levels of (a) IFN-*γ*, (b) TNF-*α*, (c) IL-1*β*, (d) IL-6, (e) NF-*κ*B, and (f) Nrf2 in the colorectum (Experiment 2) as determined by quantitative real-time RT-PCR. Feeding with crocin significantly decreased the expression levels of IFN-*γ* (100 and 200 ppm), TNF-*α* (100 and 200 ppm), IL-1*β* (100 and 200 ppm), IL-6 (100 and 200 ppm), and NF-*κ*B (100 and 200 ppm), compared with the AOM and DSS group. On the other hand, the mRNA expression of Nrf2 was significantly increased by the treatment with crocin (100 and 200 ppm). The expression was normalized to the *β*-actin mRNA expression. Samples were analyzed in triplicate. Data are the means ± SD from three independent assays (*n* = 5 from each treatment group). The ordinates are the relative mRNA expression levels (/*β*-actin) versus the 1.5% DSS group. *P < 0.05, **P < 0.01, ***P < 0.001 versus the 1.5% DSS group.

Figures 9 and 10 show the relative mRNA expression levels of COX-2 (Figure 10(a)), iNOS (Figure 10(b)), IFN-*γ* (Figure 11(a)), TNF-*α* (Figure 11(b)), IL-1*β* (Figure 11(c)), IL-6 (Figure 11(d)), NF-*κ*B (Figure 11(e)), and Nrf2 (Figure 11(f)), when the value of the DSS alone group was converted to 100 by an RT-PCR analysis. The expression levels of all of these genes except for Nrf2, in the mice treated with DSS were increased in comparison with the mice treated with 200 ppm crocin. Feeding with crocin at 200 ppm significantly decreased the mRNA expression of all of the molecules except for Nrf2. As to Nrf2 (Figure 11(f)), With regard to Nrf2, the mRNA expression level in the DSS alone group was the lowest, and the crocin treatment increased its expression. 

## 4. Discussion

We demonstrated that dietary crocin, which is a water-soluble carotenoid used as a colorant, effectively suppressed colitis and colitis-related colorectal carcinogenesis in mice. The suppressive effects were at least partly due to the antiinflammatory properties of the crocin (as indicated by the inhibition of several cytokines and inducible inflammatory enzymes). Crocin, isolated from saffron, was previously shown to inhibit the growth of several human cancer cell lines [1], including colorectal cancer cells [22]. Crocin also inhibited mouse skin [26, 28] and liver carcinogenesis [29], but there have so far been no reports of its effects on other tissues. This is the first report to show evidence that crocin inhibits colorectal carcinogenesis in rodents.

In general carotenoids possess potent cancer chemopreventive properties [54]. Major clinical trials using high-dose supplemental *β*-carotene were performed, because it is the carotenoid most frequently identified to have a protective activity against lung cancer, but it failed to demonstrate a sufficient protective effect [54]. These findings suggest that the use of carotenoids without the potential for conversion to vitamin A may provide protection and avoid this toxicity. In this study, we did not observe any clinical or histopathological toxicity of crocin. Therefore, crocin is of considerable interest because of its potent antiinflammatory, anticarcinogenesis, and antioxidant activities, which are distinctly different from those of *β*-carotene and other carotenoids [55, 56]. We also have confirmed that crocin has stronger antioxidant activity compared to *α*-tocopherol [57]. We have also recently reported the cancer chemopreventive ability of a marine carotenoid, astaxanthin, in an AOM/DSS model [40].

In this study, the dietary administration of three concentrations of crocin ameliorated AOM/DSS-induced colonic proliferative lesions in mice. This suppression was prominent in mice treated with both 100 and 200 ppm crocin. Furthermore, the dietary crocin suppressed the proliferation activity in adenocarcinomas. These findings are in agreement with those of previous reports showing the antiproliferative effects of crocin [22, 58, 59]. Many studies have shown a variety of pharmacological effects of crocin [22, 58–61]. Among the mechanisms underlying its biological actions, the antioxidant activity was thought to be responsible for the various pharmacological effects of crocin [10, 12]. 

 The antiinflammatory effects of crocin are suggested to be based on its antioxidant activity [10, 12]. In the current study, we further examined the effects of crocin on DSS-induced colitis in mice (Experiment 2). The dietary feeding of crocin significantly suppressed several inflammatory events and NF-*κ*B expression in the colorectal mucosa of the mice that received DSS. Inflammatory genes, such as COX2, iNOS, TNF-*α*, and IL-1*β*, are the most common target genes participating in the activation of NF-*κ*B and are associated with a number of chronic inflammatory diseases, including IBD and IBD-related colorectal carcinogenesis [37, 38, 45, 46, 62]. In the current study, we observed decreases in the mRNA expression levels of NF-*κ*B, COX-2, iNOS, TNF-*α*, IL-1*β*, and IL-6 in the mice treated with DSS and crocin when compared to the mice given DSS alone. Our findings thus suggest that crocin suppressed the mouse colonic inflammation induced by DSS by modulating the NF-*κ*B signaling pathway. The NF-*κ*B signaling pathway also has a major role in inflammation-associated carcinogenesis [63]. Therefore, NF-*κ*B is a target for cancer chemoprevention [42, 44], and natural compounds that suppress NF-*κ*B expression may be useful for cancer chemoprevention [64].

Although we mainly discussed the effect of crocin on the NF-*κ*B pathway, the effects of dietary crocin on Nrf2 expression of adenocarcinoma cells and the inflamed colon are also of interest, because of the protective role of Nrf2 against the toxicity of xenobiotics [47, 48]. In this study, the immunohistochemical expression of Nrf2 was increased by crocin treatment (Experiment 1). In the inflamed colon exposed to DSS, crocin increased the mRNA expression of Nrf2. Although the exact mechanism(s) underlying the elevation of Nrf2 expression by crocin treatment need to be elucidated, this elevation might partly contribute to the inhibition of colitis and colitis-related colorectal carcinogenesis by feeding with crocin. Our findings also support that Nrf2 is one of the targets for cancer chemoprevention [47–50]. 

In this study, crocin inhibited DSS-induced colitis by directly affecting the absorption of DSS. It has been reported that intestinal microflora played important role in DSS-induced intestinal inflammation [65]. Since many plant extracts were reported to benefit intestinal microflora, it may be possible that alterations in intestinal microflora by crocin feeding contribute to the antiinflammatory effects of crocin in mice that received DSS. Further studies are necessary to assess the role of crocin in intestinal microflora in a colitis-associated colorectal carcinogenesis model which was used in this study.

Taken together, the results of the present study suggest that dietary crocin inhibits AOM/DSS-induced colitis-associated colon carcinogenesis and DSS-induced colitis in mice by suppressing the expression of cytokines including NF-*κ*B. Our findings indicate that the NF-*κ*B signaling pathway may also play an important role in colitis-associated colorectal carcinogenesis and is a potential target for colitis-related colorectal carcinogenesis. Our data also suggest that crocin is a potentially effective cancer chemopreventive agent that can be used to prevent the development of CRC in the inflamed colon. Importantly, crocin demonstrated negative results in bacterial tests for mutagenicity and did not produce any chromosome damage in mammalian cells in culture [30], thus suggesting that clinical trials of crocin may be possible.

## Figures and Tables

**Table 1:** Body and liver weights and the length of large bowel of mice at wk 18.

Group no.	Treatment	No. of mice examined	Body wt (BW, g)	Liver wt (g)	Relative liver wt (g/100 g BW)	Length of large bowel (cm)
1	AOM/DSS	20	44 ± 5^a^	2.26 ± 0.37	5.19 ± 0.72	12.6 ± 1.4
2	AOM/DSS/50 ppm crocin	20	47 ± 6	2.31 ± 0.46	4.90 ± 0.58	12.7 ± 1.0
3	AOM/DSS/100 ppm crocin	20	47 ± 4	2.35 ± 0.39	5.00 ± 0.88	12.8 ± 1.2
4	AOM/DSS/200 ppm crocin	20	45 ± 4	2.20 ± 0.31	4.95 ± 0.74	12.8 ± 1.5
5	200 ppm crocin	10	42 ± 5	1.99 ± 0.31	4.74 ± 0.66	12.1 ± 1.4
6	None	10	42 ± 4	1.95 ± 0.18	4.70 ± 0.27	12.6 ± 0.9

^a^Mean ± SD.
